# Effectiveness of integrated care on delaying chronic kidney disease progression in rural communities of Thailand (ESCORT study): rationale and design of the study [NCT01978951]

**DOI:** 10.1186/1471-2369-15-99

**Published:** 2014-06-25

**Authors:** Teerayuth Jiamjariyaporn, Atiporn Ingsathit, Kriang Tungsanga, Chatri Banchuin, Kotcharat Vipattawat, Suphattra Kanchanakorn, Vinai Leesmidt, Watcharapong Watcharasaksilp, Akhathai Saetie, Chanida Pachotikarn, Sunard Taechangam, Tanyarat Teerapornlertratt, Teerachai Chantarojsiri, Visith Sitprija

**Affiliations:** 1 Bhumirajanagarindra Kidney Institute, Bangkok, Thailand; 2 Division of Nephrology, Ramathibodi Hospital, Mahidol University, Bangkok, Thailand; 3 Division of Nephrology, Faculty of Medicine, Chulalongkorn University, Bangkok, Thailand; 4 Khlong Khlung District Hospital, Ministry of Public Health, Khamphaeng Phet, Thailand; 5 Saithong Wattana District Hospital, Ministry of Public Health, Khamphaeng Phet, Thailand; 6 Institute of Nutrition, Mahidol University, Bangkok, Nakhon Pathom Province, Thailand; 7 Queen Saovabha Memorial Institute, Bangkok, Thailand

**Keywords:** Chronic kidney disease, Integrated CKD care program, Village health volunteers

## Abstract

**Background:**

In developing countries, accessibility to specialists, and physician to patient contact time is limited. In Thailand, A unique community health service is provided by subdistrict health care officers and Village Health Volunteers (VHVs). If the personnel were trained on proper chronic kidney disease (CKD) care, CKD progression would be delayed.

**Methods/Design:**

We conducted a community-based, cluster randomized controlled trial at Kamphaeng Phet Province, located about 400 kilometers north of Bangkok. Two out of eleven districts of the province were randomly selected. Approximatly 500 stage 3–4 CKD patients from 2 districts were enrolled. Patients in both groups will be treated with standard guidelines. The patients in intervention group were provided the additional treatments by multidisciplinary team in conjunction with community CKD care network (subdistrict health care officers and VHVs) which will provide group counseling during each hospital visit and quarterly home visits to monitor dietary protein and sodium intake, blood pressure measurement and drug compliance. Duration of the study is 2 years. The primary outcome is the difference of rate of eGFR decline. The secondary outcomes are laboratory parameters and incidence of clinical endpoints such as mortality rate and cardiovascular events, end-stage renal disease (ESRD), etc.

**Discussion:**

Insights of this study may set forth a new standard of community-based CKD care.

**Trial registration:**

NCT01978951.

## Background

According to a recent community-based survey among Thais, the prevalence of pre-dialysis (stage 3, 4 and 5) CKD was 8.8% [1]. It is estimated that the total number of stage 3–4 CKD patients in Thailand is 4.1 millions. With only 450 active nephrologists, the ratio of CKD patients to nephrologists in Thailand is as high as 1:15,000. The high ratio indicates that accessibility to nephrologist may not be equal for all CKD patients. At present, Thai CKD care is still based on conventional method in which a hospital-based, physician-oriented approach provided at the out-patient department [2-4]. In rural areas where physician-to-patient contact time is more limited and renal specialists are scarce or not existing, this kind of health care service is impractical. Thus, it is mandatory to seek other appropriate forms of renal care to delay CKD progression.

Thailand is geographically divided into 77 provinces. Each is divided into 5–10 districts and further into 5–10 subdistricts per district. In general, a typical subdistrict consists of 10–15 villages, each consists of 100–150 households. At the district level, there is one district hospital consisting of 3–5 general practioners providing all basic medical services including maternal and child health care, simple trauma, diabetes and hypertension. There is one subdistrict health care office in each subdistrict, with 1–2 nurses, 3–5 subdistrict health care officers, and 100–120 village health volunteers (VHVs). Healthcare personnel of each subdistrict involved in various community health activities, for example, anti-smoking campaign, HIV prevention, etc.

The VHVs are villagers voluntarily recruited from their own villages. Each VHV is responsible for 10–15 households. They work as a bridging point between villagers and health care personnel at the subdistrict health offices or district hospitals. At the moment, there are about 980,000 VHVs covering more than 90% of all villages in Thailand. With continued and unrelenting contact between VHVs and their respective household villagers, the intimate bonding” and “sense-of-belonging” relationship is created. The VHV scheme serves as the backbone of community-based public health service in Thailand. This system is so successful that Thailand is regarded as one of a few developing countries that have efficient primary health care service [5,6].

Should the paramedical personnel be trained to render proper CKD care, it would be interesting whether their intimate relationship and commitment to their responsible village households will result in better outcome when compared with the conventional care. The aim of this study is to compare the effectiveness on delaying CKD progression between a conventional care and an integrated CKD care provided by multidisciplinary team of hospital staffs in conjunction with the community CKD care network (subdistrict health care officers and VHVs).

## Methods

### Study design

**Figure 1:**
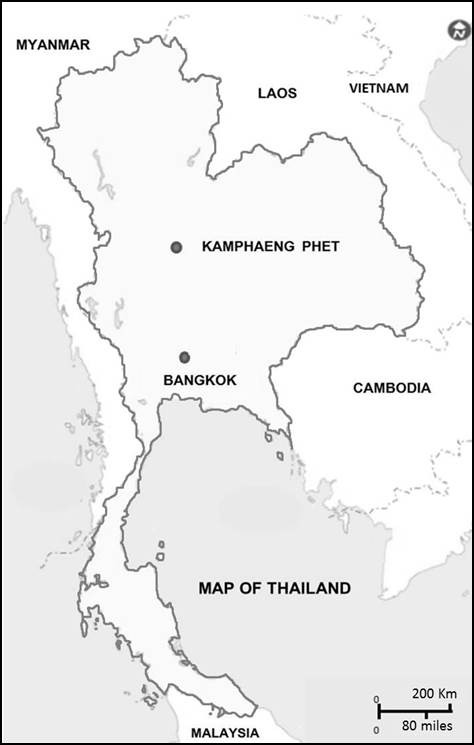
Map of Thailand.

**Figure 2:**
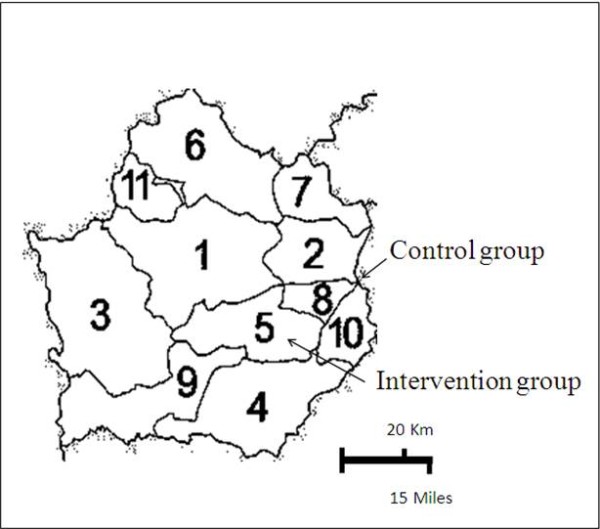
**Map of Kamphaeng Phet Province.** The numbers represent respective districts of the Province. District No.5 and No.8 denote Khlong Khlung District (Intervention group) and Sai Thong Wattana District (Control group), respectively.

The ESCORT study is a community-based, cluster randomized controlled trial. Duration of the study is 2 years. This study has been conducted at Kamphaeng Phet Province, located about 400 kilometers north of Bangkok (Figure 1 and Figure 2). Due to strong relationship among neighboring households in rural communities, it is unable to assign subjects living in the same district to be randomly received either of the 2 CKD care programs without crossover interference from one subject in one household to another. As a result, we have conducted a cluster randomized controlled trial. Two out of eleven districts of the Province, namely Sai Thong Wattana District (control group) and Khlong Khlung District (intervention group), were randomly selected. Details of Public health characteristics of these 2 districts were described in Table 1.

**Table 1:** Public health data of the 2 districts under the study

	**Control group**	**Intervention group**
Total population	23,821	73,374
Occupation: farmer (%)	80	80
No. of subdistricts	3	10
No. of villages	38	103
No. of personnel at the district hospitals		
• General practitioners	3	5
• Nurses	25	54
• Pharmacists	2	5
• Nutritionists	0	1
No. of personnel at each subdistrict health office		
• Nurses	1	1
• Subdistrict health care officers	3-5	3-5
No. of village health volunteers per village	10-12	10-12

### Enrollment

The study was approved by the Ethics Committee of Institutional Review Board of Ministry of Public Health, Thailand and was registered with http://www.clinicaltrials.gov (NCT01978951). Written inform consents were obtained prior to recruitment. The study was conducted in accordance with good clinical practice and the Declaration of Helsinki [7]. Eligible cases are those who live in these 2 districts, age of 18–70 years old and had diabetes and/or hypertension. Patients whose eGFR was in a range of 15–59 ml/min/1.73 m^2^ body surface area estimated twice at 3 months apart were asked to participate in this study. Creatinine-based estimated glomerular filtration rate (eGFR) was calculated with the CKD-EPI equation [8]. Patients were excluded if they have any of the following: unstable/advanced cardiovascular disease, obstructive uropathy, ESRD, HIV infection, pregnancy, body mass index (BMI) less than 18 or more than 40 kg/m^2^, being under treatment for malignancy, urine protein-creatinine ratio more than 3.5 g/g creatinine and active urinary sediment (urine red blood cells more than 3 cells/high power field or urine white blood cells more than 10 cells/high power field). Hypertension was defined as a systolic blood pressure higher than 140 mmHg or a diastolic blood pressure higher than 90 mmHg or concomitant use of antihypertensive medications [9]. Blood pressure was recorded twice with a sphygmomanometer with 15-minute rest between the measurements. Diabetes was diagnosed when a fasting plasma glucose is at or above 126 mg/dl or HbA1C at or above 6.5 percent twice after repeated measurements [10].

### Sample size

In a previous observational study, the rate of eGFR decline among diabetic patients with overt nephropathy was 4.2 ± 3.8 ml/min/1.73 m^2^ BSA per year [11]. We hypothesized that if the rate of eGFR decline of intervention group is less than control group at least 1 ml/min/1.73 m^2^ per year, the primary outcome of the study will be interpreted as significant. To acheive 80 percent or higher power to detect the difference between the two treatments, it was estimated that 228 stage 3–4 CKD patients were needed to be enrolled to each group [12]. Taking into account that 15% of patients might be loss to follow-up because of non–end point death or other reasons, approximately 250 patients for each group were required.

According to the inclusion and exclusion criteria, recruitment had done in January to June 2011. About 25% of candidates were eligible and asked to participate in the study. The main reason for non-participation was inability of the patient and caregiver to attend the anticipated visits. In July 2011, 433 patients had been enrolled . Enrolled subjects of each group will be further subdivided into 12 patient subgroups with respect to the vicinity of their subdistrict. Each subgroup will consist of 25–30 CKD patients.

### Interventions

**Figure 3:**
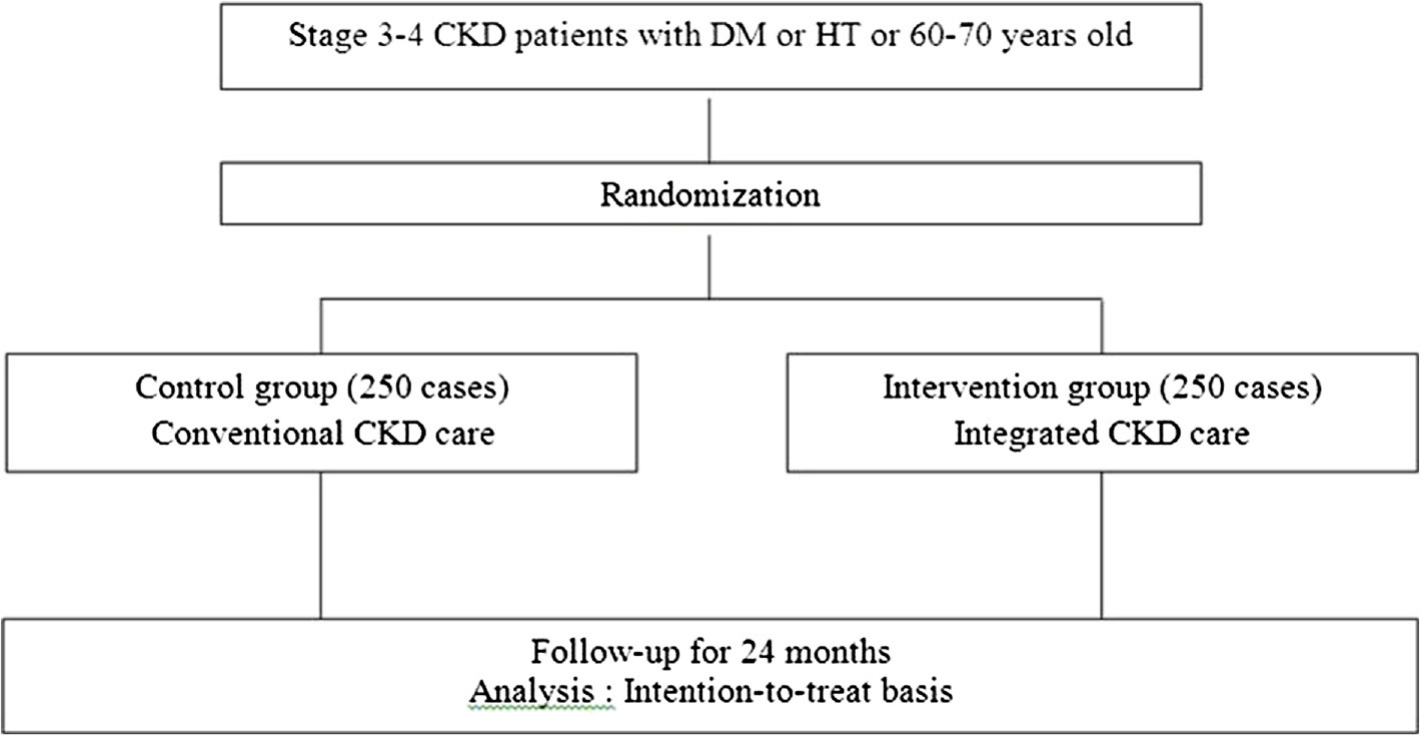
Design of the study.

Two types of CKD care programs will be provided separately according to their respective district of residence. CKD patients residing in district No.8 will be assigned to the control group and those in district No.5 will be assigned to the intervention group (Figure 3). It should be noted that medical facilities in these 2 district hospitals are quite comparable (Table 1). Three investigators who are nephrologists [T.J., K.V., S.K.], will provide essential knowledge about CKD care and treatments according to NKF-K/DOQI guidelines to all staffs of both groups [9].

In the control group, two general practitioners and two chronic care nurses will provide CKD care to the subjects according to treatment guidelines (Table 2). All medical cares and educational programs will be provided during their visit to the district hospital. Educational activities will be provided as group counseling. The educational topics will be arranged sequentially i.e. basic knowledge about CKD, optimal diet for pre-dialysis CKD patients, effect of hypertension on CKD, relationship between diabetes mellitus and CKD, drug use in CKD, and renal replacement therapy.

**Table 2:** **Treatment guidelines for both treatment groups **[8]

**Clinical and laboratory parameters**	**Treatment targets**
Blood pressure	Less than 130/80 mmHg
Body mass index	18.5-24.9 kg/m^2^
Hemoglobin	More than 10 g/dl
HbA1C	Less than 7%
LDL-cholesterol	Less than 100 mg/dl
Serum bicarbonate	More than 22 mEq/L
Urine protein-creatinine ratio	Less than 500 mg/g
Sodium intake	Less than 2,400 mg/day
Protein intake	Less than 0.8 g/kg ideal body weight /day
Smoking	Smoking cessation
Physical exercise	30 minutes or more per day 3 times a week

#### ***Integrated CKD care program***

In the intervention group, an integrated CKD care program (ICP) will be implemented. The ICP is a collaborative CKD care program. The key process is the cooperation between multidisciplinary team (MDT) and community CKD care network (CCN). The MDT consists of two general practitioners, two chronic care nurses, one pharmacist, one nutritionist, and one physical therapist. Each CCN subgroup consists of 1 subdistrict health care officer, 3–5 VHVs, and family members of the CKD patients residing in the respective patient subdistrict.

In the pre-study period, a 4-day CKD care training-course will be organized for members of CCN before commencement of the intervention program. The training-course contents include basic knowledge of CKD, NKF-K/DOQI guidelines, and optimal diet for CKD patients. The contents has been simplified to match with their basic medical knowledges. Two dietitians [C.P., S.T.] will provide essential knowledge about collection and interpretation methods of modified 24-h dietary recalls or Easy Dietary Assessment tool (EDA) [13]. CCN members must pass Pre/Post test evaluation with a minimum score of 75 percent preceeding commencement of the program.

After all staffs were completely trained, the MDT will provide educational activities as live demonstration of optimal diets, medication, and exercise for CKD patients during each hospital visit in addition to standard treatments. Meanwhile, each subgroup of CCN will provide home visits to their respective patients at 6–8 weeks after each hospital visit. The activities during each home visit will include the 4 main following issues: 24-h dietary recalls, blood pressure measurement, medication compliance monitoring including avoidance of nephrotoxic agents (e.g. NSAIDs), exercise monitoring, and 24-h dietary recall. The CCN will ask 24-h dietary intake and will use EDA tool to estimated dietary protein and sodium intake and feedback the results to their patients during each home visit. Details of clinical intervention during hospital and home visits of both groups were illustrated in Table 3.

**Table 3:** Intervention of the study

**Treatments**	**Control group**	**Intervention group**
**Standard CKD care according to NKF-K/DOQI guidelines**	**Yes**	**Yes**
Multidisciplinary team provide educational activities during each hospital visit.	No	Yes
Topics:		
1. Optimal diets for CKD patients		
2. CKD medications		
3. Exercise for seniors		
Community CKD care network provide home visit.	Yearly	Quarterly
Key activities:		
1. 24-hour dietary recall		
2. Blood pressure measurement		
3. Medication compliance monitoring		
4. Exercise monitoring		

### Clinical and laboratory assessments

Patients in both groups will be scheduled to follow-up at their respective district hospitals at every 3 months. During each hospital visit, blood pressure, body weight, and waist circumference will be assessed. Laboratory measurement will be done at enrollment, the first month and every 3 months thereafter or when patients are withdrawn from the study (Table 4). Blood pressure will be recorded twice with a sphygmomanometer with a 15-minute rest interval. The ACEi/ARBs dosages will be adjusted by the caring physicians. In case of any patient develops intolerable side-effects of ACEi/ARBs, other antihypertensive agents will be substituted.

**Table 4:** Schedule of clinical and laboratory assessments during the study period

**Visit**	**Screening period**	**Visit 1**	**Visit 2**	**Visit 3**	**Visit 4**	**Visit 5**	**Visit 6**	**Visit 7**	**Visit 8**	**Visit 9**	**Visit 10**
**Month**		**0**	**1**	**3**	**6**	**9**	**12**	**15**	**18**	**21**	**24**
Blood pressure, body weight, waist circumference	√	√	√	√	√	√	√	√	√	√	√
Serum creatinine	√	√	√	√	√	√	√	√	√	√	√
eGFR	√	√	√	√	√	√	√	√	√	√	√
Serum potassium	-	-	√	-	√	-	√	-	√	-	√
Phosphate, Calcium, Albumin	-	√	-	-	√	-	√	-	√	-	√
Hemoglobin	-	√	-	-	√	-	√	-	√	-	√
HbA1C	-	√	-	-	√	-	√	-	√	-	√
LDL-cholesterol	-	√	-	-	√	-	√	-	√	-	√
Urine dipstick protein	-	√	-	-	√	-	√	-	√	-	√
Urine protein -creatinine ratio	-	√	-	√	√	√	√	√	√	√	√
24-hour Urine for Na, Urea-N, Creatinine, Protein	-	√	-	-	√	-	√	-	√	-	√

### Laboratory analysis

A protocol of laboratory investigation is summarized in Table 4. Prior to each hospital visit, all subjects will be instructed to have an 8-hour overnight fast. Plasma glucose, lipid profile, HbA1C, albumin, electrolytes, and urine analysis will be measured in the next morning. After centrifugation, plasma and urine samples will be centrifuged and transported at 4°C and analyzed within 24 hours at the laboratory Department of the District 2 Hospital. All blood chemistries will be measured with ABX Pentra 400 analyzer (HORIBA ABX S.A.S., France). Urine protein and creatinine will be measured with the pyrogallol red colorimetric method with the same analyzer. All biochemistry analyses will be validated according to the standard protocol of Department of Medical Sciences, Ministry of Public Health, Thailand. Serum creatinine will be measured by the enzymatic method. Serum creatinine measurement will be standardized with standard reference material (SRM 967) by commutability study at every 6 months [14].

Two out of twelve subgroups of both groups will be randomly selected and assigned to collect 24-hour urine. An aliquot of 24-hour urine of each sample will be sent for urine chemistry analysis protein, creatinine, urea, and sodium at Laboratory Department of Division of Nephrology, Chulalongkorn University, Bangkok, Thailand.

### Outcomes

The primary outcome of this study is the difference of rate of eGFR decline. The secondary outcomes are laboratory parameters (Table 4) and incidence of clinical endpoints including all-cause mortality, cardiovascular events (acute myocardial infarction and stroke), ESRD (eGFR is less than 15 ml/min/1.73 m^2^), and 50% increase in serum creatinine from baseline. In this study, acute myocardial infarction is defined as a new ischemic pattern change of ECG and significant rising of cardiac enzymes. Acute stroke is defined as there is a clinical significant neurological deficit with an objective evidence by appropriate imaging study (e.g. computerized tomography scan). Patients will be followed at the first month and every three months after enrollment. Subgroup analysis will be done for selected parameters including age, sex, and diabetes mellitus. Quality of life will be assessed using a validated questionnaire, Thai SF-36 health survey [15].

### Statistical analysis

Analysis of the primary outcome will be based on an intention-to-treat basis. Descriptive statistics will be expressed as the mean and standard deviation. Discrete variables will be presented as frequency and percentage. An independent t-test will be applied to compare mean values and mean differences between intervention and control groups. Differences between categorical variables will be analysed with Chi-square or Fisher’s exact tests. The comparison of the rate of eGFR decline will be analyzed using linear mixed models (Generalized Estimating Equation) [16]. The difference of incidence of clinical endpoints will be analysis using Cox-regression analysis. All statistical tests will be two-tailed tests. A P-value of less than 0.05 will be considered statistically significant. The data will be analysed using SPSS 16.0 (SPSS Inc., Chicago, IL, USA).

## Discussion

Currently, Thailand has a population of approximately 66 million, in which 55 percent of the population live in rural areas [17,18]. In these areas, the number of specialized personnel is limited and work burden is overwhelming, good clinical care and equal accessibility to the care may be too difficult to achieve. Utilizing renal physicians, or even internists, to take care of CKD patients in rural area is rather far from reality. To combat these problems, we try to search for an appropriate CKD care model which could provide optimal treatment to the rural population. To the best of our knowledge, this study is the first large-scale, community-based cluster randomized controlled trial to study the effect of employing an integrated CKD care which is comprised of multidisciplinary CKD care program in conjunction with well-trained paramedical personnel on delaying CKD progression.

In addition to conventional CKD treatment, the two key processes will be added into the intervention group. Firstly, the multidisciplinary team of district hospital staffs will provide demonstration about essential knowledge CKD care during each hospital visit. We hypothesized that this interactive activity will provide better understanding of essential medical knowledge and increase awareness of burden of CKD and ESRD. The second process is quarterly home visit by the CCN. We trained the CCN and endorsed them to work as the escorts (as the abbreviation name of the study) to protect CKD patients. The important roles of CCN is monitoring compliance to the treatments and instantly feedback to the patients. These important informations will be used to modify lifestyle of CKD patients and may improve compliance to the treatments.

Interestingly, the integration of both processes will increase frequency personnel to patients contact compensating for physician to patient contact time which may improve compliance of drug usage and dietary control; furthermore, the continuity of monitoring might be a key mechanism for sustainable self-behavioral modification. We expect the results of this study will set forth a new standard of community-based CKD care for Thailand.

### Limitation of the study

We conducted cluster randomized control trial instead of double-blinded randomized control trial in any district, for patients who reside in the same district may imitate the way of practice of each group of the study. Due to ethical consideration, the control group have to inevitably provide essential knowledge according to standard guidelines which may improve their rate of eGFR decline. Finally, the interventions of this study are multifactorial design, so we are unable to determine the single risk factors and need further investigation to identify determinant factor for behavioral modification.

## Abbreviations

DM: Diabetes mellitus; HT: Hypertension; eGFR: Estimated glomerular filtration rate; CKD: Chronic kidney disease; VHV: Villege health volunteers; MDT: Multidisciplinary team; CCN: Community care network.

## Competing interests

The authors declare that they have no competing interests.

## Authors’ contributions

TJ drafted the manuscript. AI contributed to study design and statistical analysis. KT and CB extensively contributed to the conceptual design of the study and helped to draft the manuscript. KV, SK, AS, CP, and ST participated in the training program of intervention group and control group. VL, WW, TT, TC, and VS participated in the study design and coordination of the study. All authors read and approved the final manuscript.

## Pre-publication history

The pre-publication history for this paper can be accessed here:

http://www.biomedcentral.com/1471-2369/15/99/prepub
